# The complexity of home-based rehabilitation technology implementation for post-stroke motor rehabilitation in the Netherlands

**DOI:** 10.1186/s12913-024-12044-2

**Published:** 2025-01-04

**Authors:** Karlijn E. te Boekhorst, Sanne J. Kuipers, Gerard M. Ribbers, Jane M. Cramm

**Affiliations:** 1 Socio-Medical Sciences Department, Erasmus School of Health Policy & Management, Erasmus University Rotterdam, Rotterdam, The Netherlands; 2 Rehabilitation Medicine Department, Erasmus University Medical Centre, Rotterdam, The Netherlands

**Keywords:** Rehabilitation technology, Implementation, Post-stroke motor rehabilitation, Stroke, Home-setting, NASSS framework

## Abstract

**Background:**

Rehabilitation technology is a growing field, but the sustainable implementation of these technologies, particularly in home settings, is lacking. The aim of this study was to explore the factors influencing the uptake of stroke rehabilitation technology among various stakeholders, including developers, healthcare professionals, individuals who had strokes, strategic experts, management and innovation staff, health insurers, and the National Health Care Institute.

**Methods:**

In total, 22 semi-structured interviews were conducted with a purposive stakeholder sample. The Non-adoption, Abandonment, Scale-up, Spread, and Sustainability (NASSS) framework was used as the theoretical basis for the interview design. The interview content was analysed to generate (sub)themes representing factors influencing the implementation of home-based rehabilitation technology. These (sub)themes were organised according to the NASSS framework domains to ensure a systematic and theoretically grounded analysis.

**Results:**

Ten influencing factors emerged, nine of which fell within six of the seven NASSS domains. These factors include: (1) the unpredictable aftermath of stroke, (2) technology (mis)alignment with care delivery processes and end users’ preferences, (3) disparities in the assessment of technology’s value, (4) differences in commercial and university developers’ interests, (5) patient group capabilities, (6) perceived workload, (7) formal implementation plans in rehabilitation centres, (8) laws and regulations, and (9) the financial system. The factor that did not align with a single NASSS domain was: (10) the fragmentation of responsibilities among diverse stakeholders.

**Conclusion:**

This study shows that the sustainable implementation of home-based rehabilitation technology faces several challenges across multiple domains of the NASSS framework. Effective collaboration among stakeholders is crucial for addressing these challenges but is currently hindered by fragmented responsibilities. To improve collaboration, it is essential to clearly define the roles and responsibilities of all stakeholders. Additionally, national-level policies adopting a systems approach are necessary to align these responsibilities and foster effective collaboration.

**Supplementary Information:**

The online version contains supplementary material available at 10.1186/s12913-024-12044-2.

## Background

In the Netherlands, stroke incidence has remained stable from 2018 to 2023 [1] but is expected to rise due to an ageing population [2]. Meanwhile, mortality rates have decreased from 166.2 per 1,000 in 1980 to 52.3 per 1,000 in 2022 due to advancements in stroke care [3], such as early thrombolysis and specialised stroke units [4]. This reduction in mortality, coupled with the expected rise in stroke incidence, will likely drive up the demand for rehabilitation services, which currently account for 1.4% of total healthcare expenditure [5]. To meet this increasing demand and at the same time limit the growth of stroke rehabilitation costs, more efficient rehabilitation strategies are needed.

Rehabilitation technology, a rapidly growing field [6, 7], seems to be a promising tool for creating such strategies. Research has identified multiple benefits of these technologies: they can reduce stroke-related motor impairments [8–14], enhance the intensity of therapy without the need for additional direct therapist–patient contact time, motivate patients to engage in therapy through gamification, support the practice of relevant daily activities (e.g., sandwich making, laundry folding, and grasping objects from a distance) [6, 9, 12–14], and reduce the workload of healthcare professionals [15]. To date, rehabilitation technology has primarily been implemented in clinical settings [12, 14, 15]. However, the Dutch national Integrated Care Agreement (in Dutch: *Integraal Zorg Akkoord*) emphasises the importance of providing care in the most appropriate setting [16]. Consequently, the agreement promotes transitioning rehabilitation services to non-clinical environments, such as patients' homes, where suitable. This shift positions home-based rehabilitation supported by technology as an increasingly viable option for stroke rehabilitation.

Despite its potential, the implementation of home-based rehabilitation technology has proven to be challenging [17, 18]. Contributing factors include stroke survivors' abilities and willingness to use the technology at home [15, 19, 20], healthcare professionals' concerns about safety, time constraints, and limited expertise [15, 20–22], insufficient attention to user preferences during development [18], and the absence of reimbursement mechanisms [23]. Addressing these factors is crucial for the successful adoption and sustainable implementation of home-based rehabilitation technologies.

However, earlier research on the implementation of home-based rehabilitation technology has narrowly focused on certain types of technology or stakeholders [15, 18–23]. This focus has resulted in a fragmented understanding of the full range of factors that influence the implementation process, such as the needs and views of key stakeholders like health insurers and rehabilitation centre managers. This fragmentation hinders the ability to attain a complete overview of the involved factors and their interactions, potentially leading to a suboptimal approach to implementation and, consequently, to less effective outcomes. Thus, a more holistic approach is required. This involves adopting a systems-thinking perspective, which considers the interdependencies among all relevant stakeholders and their roles within the broader system. Such a systemic approach can help identify and address the complex factors influencing implementation and support its long-term sustainability [24, 25]. Accordingly, the aim of this study is to apply a systems-thinking approach to explore the factors influencing the uptake of home-based rehabilitation technology for post-stroke motor rehabilitation.

### Theoretical framework

This study uses the Non-adoption, Abandonment, Scale-up, Spread, and Sustainability (NASSS) framework, developed by Greenhalgh et al. [26], to examine the factors that affect the implementation and sustainability of home-based rehabilitation technologies. The NASSS framework is designed to address the challenges of introducing technology-supported health and care programmes from a systems perspective. It consists of seven domains: condition, technology, value proposition, adopter system, organisation, wider system, and embedding and adaptation over time [26]. These domains are based on a combination of theories from different disciplines, including socio-technical theories, technology adoption, normalisation process theory, and user-centred design. The framework was also shaped by empirical data collected from ethnographic observations and semi-structured interviews, ensuring its practical relevance [26]. A full overview of these domains is provided in Table 1.

**Table 1:** The seven domains of the NASSS framework [26]

Domain	Description
Condition	The health condition or illness targeted by the technology, including its clinical aspects, related comorbidities, and relevant sociocultural factors
Technology	The technology being implemented, encompassing both hardware and software elements
Value proposition	The value generated by the technology, including supply-side value (for developers) and demand-side value (for patients, healthcare professionals, healthcare organisations, insurers, and the health system)
Adopter system	The end-users of the technology, focusing on their willingness and ability to use it
Organisation	The cultural and organisational characteristics of the involved organisations, such as their structure, capacity to implement new practices, and the availability of skilled staff and resources
Wider system	The national and local contexts influencing the implementation of technology, including the political system (e.g., laws and regulations), economic system (e.g., financial infrastructure), professional environment (e.g., medicolegal standards), and sociocultural context (e.g., public interest and expectations)
Embedding and adaptation over time	The key changes expected over the next 3–5 years that could influence the implementation of the technology

The NASSS framework is widely used as a structured approach to analysing healthcare technologies. Literature reviews have confirmed its validity and relevance across various healthcare contexts [27, 28]. These reviews demonstrated how the NASSS framework helps in identifying multi-level factors affecting the implementation of health technologies, revealing important contextual details, and clarifying the relationships between the health system, its environment, and the stakeholders involved [27, 28]. Given its systems approach to implementation, specific focus on health technologies, and proven effectiveness across healthcare contexts, the NASSS framework is well-suited to guide both data collection and analysis in this study.

## Method

### Study design

Using a phenomenological qualitative research design [29], we conducted semi-structured interviews with 22 stakeholders involved in post-stroke rehabilitation. This type of data collection contributes to a better understanding of stakeholders’ attitudes and expectations [30]. The NASSS framework [26] was used as a theoretical basis and analytical lens to extrapolate the multiple factors that influence the implementation, scale-up, spread, and sustainability of home-based rehabilitation. The COREQ guidelines were followed to ensure adequate study design and reporting [31].

### Recruitment and inclusion

A purposive sample of stakeholders was recruited for this study. Stakeholders were selected based on their expertise, roles, or influence on the implementation process: individuals who had a stroke and healthcare professionals with direct post-stroke rehabilitation experience were chosen for their practical insights; management and innovation staff from rehabilitation centres were included for their role in technology implementation; technology developers were selected for their design expertise; health insurers for their role in reimbursement decisions; a representative from the National Health Care Institute was included for their advisory function on reimbursement; and strategy experts were selected for their thorough understanding of the healthcare field.

To identify and recruit these stakeholders, we employed various approaches. The individuals who had a stroke were identified through the personal network of the first author (KB). They were initially contacted via WhatsApp to provide an overview of the study and invite them to participate in interviews. This was followed by a phone call to provide a detailed explanation and schedule the interview. To ensure their well-being, participants could have an informal caregiver present, and the interviewer (KB) was trained to recognise signs of discomfort, allowing for pauses or termination of the interview if necessary. The other stakeholders were recruited from the professional networks of the authors (KB, GR, and JC). The first author (KB) contacted them via email to outline the study and invite them to participate in interviews. Additionally, snowball sampling was employed, with the initial participants recommending other relevant individuals.

### Data collection

From November 2023 to January 2024, the first author (KB) conducted semi-structured interviews in Dutch, with an average duration of 52 min. Interviews with individuals who had a stroke were conducted at their homes, with their informal caregivers present. Interviews with the other stakeholders were conducted either online or at their workplaces, depending on their preferences. These interviews involved only the interviewer and the participant. We developed an interview guide (Supplementary File 1) for this study, following the methodology proposed by Kallio et al. [32]. This guide is based on the descriptions of the domains of the NASSS framework: condition, technology, value proposition, adopter system, organisation, and the wider system [26]. The questions were tailored to suit the respective stakeholders' needs and expertise. The guide was reviewed after the first interview, but no changes were made. Written informed consent and permission to audio record the interviews were obtained from all participants. No field notes were taken during the interviews.

### Data analysis

Qualitative content analysis of the interview data was performed as described by Graneheim and Lundman [33]. Initially, all interview audio recordings were transcribed verbatim. The first author (KB) reviewed the transcripts multiple times to gain a comprehensive understanding of the content before importing the transcripts into Atlas.ti (version 23.1.1) [34]. Data analysis, including coding and (sub)theme extraction, was performed using this software.

The analytical process involved several stages. First, based on the study objectives, the significant statements (meaning units) that related to the participants' perspectives on the factors influencing the implementation of home-based rehabilitation technology were identified, and each meaning unit was given a code (Table 2). Second, the codes were grouped into (sub)themes based on their conceptual similarities and differences. Third, the (sub)themes were compared and grouped into the domains of the NASSS framework. The initial coding was carried out by the first author (KB), and the aggregation of codes into (sub)themes within the NASSS framework was refined through iterative discussions with the second (SK) and fourth (JC) authors (Supplementary File 2). The (sub)themes within the NASSS framework were reviewed by an engineering expert in motor rehabilitation technology and a rehabilitation medicine expert. Both experts confirmed the validity of the identified (sub)themes, and no additional (sub)themes emerged.

**Table 2:** Examples of meaning units and codes

*Meaning Unit*	Code
*‘It is a group that often has cognitive problems, which means they may not pick things up as quickly as we do. Therefore, implementing home-based rehabilitation technology can be more challenging for this patient group.’*	Complex and unique needs of individuals who had a stroke
*‘We [innovation and management staff] do not set our objectives in advance. Instead, we actively listen to understand what is happening during the implementation process and then focus on what is most important at that moment.’*	Implementation plan
*‘For example, we [health insurer] had a specific arrangement for a pilot programme in geriatric rehabilitation because [implementation of innovation] can be quite challenging, even for experienced institutions. This special arrangement allowed them to conduct a thorough investigation into which innovation is best suited for the institution and how to implement it effectively.’*	Reimbursement rehabilitation technology

### Ethical approval

Ethical approval for this study was granted by the Internal Review Board of the Erasmus School of Health Policy & Management, Erasmus University Rotterdam (no. ETH2223-0579). Before being interviewed, participants received and signed an informed consent form.

## Results

### Sample characteristics

A total of 22 individuals (mean age, 46 years; range, 24–72 years) participated in the study; the stakeholder groups comprised 1–5 participants (Table 3). Men predominated in the group of healthcare professionals. The genders were more balanced in the other stakeholder groups.

**Table 3:** Stakeholders who participated in semi-structured interviews (*N* = 22)

Stakeholder	Gender	N
People who had strokes	1 M / 1 F	2
Health insurers (e.g. innovation advisors, innovation managers)	3 M / 2 F	5
Healthcare professionals - 2 occupational therapists - 3 physical therapists	4 M / 1 F	5
Management and innovation staff of rehabilitation centres - 1 innovation manager - 1 strategical innovation advisor - 1 innovation programme manager	1 M / 2 F	3
Strategy experts - 1 professor - 1 professor, Applied Sciences - 1 strategist, sustainable healthcare	2 M / 1 F	3
Technology developers - 2 commercial technology developers - 1 university technology developer	2 M / 1 F	3
National Health Care Institute representative - medical advisor	1 F	1

### Interview content analysis results

This section describes stakeholders' views on the implementation of rehabilitation technology in the home setting. The results are reported according to the following NASSS domains: condition, technology, value proposition, adopter system, organisation, and wider system. In addition, fragmented responsibilities were observed across multiple domains. These fragmented responsibilities are discussed separately to provide a clear understanding of their impact on the implementation process. An overview of the key findings is provided in Supplementary File 2.

#### I. The condition

This domain addresses clinical, comorbidity-related, and socio-cultural aspects of the medical condition. Table 4 includes representative quotes from this domain. Healthcare professionals and developers highlighted challenges in technology development and implementation, primarily due to the unpredictability of the aftermath of stroke. Healthcare professionals noted that individuals who have had strokes may face various hurdles, including impaired motor function, cognitive decline, and language incapacities *(Q1A, B).* They specified that those experiencing cognitive decline after a stroke may find it challenging to learn new skills and retain crucial information needed for technology use at home. Tasks such as remembering the exercises, identifying the technology needed, gathering the setup information, and executing the setup present significant challenges, especially given the slower learning pace observed in this group (*Q1C*, *D*). The professionals also emphasised patient training challenges related to individuals’ diverse cultural backgrounds or aphasia (*Q1E*)*.*

**Table 4:** Selected stakeholder quotations regarding the condition

Identifier	Quote
*1A*	*'What makes the group I work with [people who have had strokes] complex is that it [stroke aftermath] involves both cognitive and motor problems and sometimes language problems. That combination can be very challenging.’ Occupational therapist A*
*1B*	*‘I see many difficulties [in developing technology for people who have had strokes]. We don’t fully understand what a stroke is (…) Developing for stroke is very challenging, as there’s potentially a cognitive problem University technology developer*
*1C*	*‘It’s important to remember that this [people who have had strokes] is a group that’s often dealing with cognitive problems, so they might not grasp things as quickly as you and me.’ Physical therapist A*
*1D*	*‘It takes quite a bit of your cognitive abilities to see, “Hey, I have to do these exercises, I have to use this device, have to sit in this position, and have to grasp this information”. There are a lot of steps.’ Occupational therapist B*
*1E*	*‘When there’s a language problem, it [patient training] is always difficult, whether that’s due to a different cultural background or conditions like aphasia. Because the instruction that I give will never come across 100%.’ Occupational therapist B*

#### II. Technology

The statements of healthcare professionals, strategic experts, and management and innovation staff members within this domain were primarily concerned with the functionality of the device. These statements are presented in Table 5. They noted that many developed technologies did not align with the actual delivery of care. For instance, sessions with healthcare professionals typically last for 30 min, and the time required for setup or interpretation of results (e.g., sensor feedback) is often substantial. This factor may negatively impact adoption, as it reduces direct patient–provider contact (*Q2A*)*.* Moreover, these stakeholders noted that the design of many technologies overlooks the specific needs and challenges of the end-user. Management and innovation staff members and strategic experts attributed this oversight to a lack of firsthand experience or knowledge of the end-users’ characteristics, which could result in the creation of solutions that, although technically proficient, do not effectively address end-users’ practical requirements and preferences (*Q2B*)*.* These stakeholder groups placed responsibility for the misalignment on developers. Developers acknowledged the knowledge gap and stressed the importance of co-designing technology with healthcare professionals (*Q2C*). According to developers, such inclusion introduces a certain level of complexity, necessitating the maintenance of a delicate balance between the preferences, ideas, and suggestions of healthcare professionals and the practicality, user-friendliness, and broader acceptance of the technology (*Q2D*). Healthcare professionals, however, noted that device complexity can arise from both parties (*Q2E*)*.* The strategic experts pointed out that the engagement of healthcare professionals does not guarantee that the developed technology will fully meet the needs of patients. They highlighted the possibility that issues might arise in translation, underscoring the importance of involving them in the co-designing process (*Q2F*).

**Table 5:** Selected stakeholder quotations regarding the technology

Identifier	Quote
2A	*‘When you’re with a patient, you only have half an hour. If you spend 10 min setting up a device, the patient can only use it for 20 min, or even less, because you have to uninstall everything. So, it’s not worthwhile installing it.’ Occupational therapist B*
2B	*‘Developers have never spent half a year wandering around in a care organisation to truly experience what really happens or personally experienced what happens after the technology is provided to the target group (…) For example, the hip airbag. Although the technology itself is effective, it can only be washed at 30 degrees. However, these people [individuals at greater risk of falling] often experience incontinence issues. Washing such items at a lower temperature is impractical [meaning that it does not get clean enough]. Developers just didn’t think about this beforehand.’ Professor, Applied Sciences*
2C	*‘Involving healthcare professionals will help us think about things we probably normally wouldn’t have thought about. So, when we start developing technology, we’re probably going to miss a lot, and by showing it and discussing it [to/with healthcare professionals], we can develop something better.’ University technology developer*
*2D*	*‘We developed a hand robot that allows for pronation and supination, flexion and extension, and squeezing and releasing—all exercises suggested by a healthcare professional. However, introducing these movements into one robot makes it a very complex robot (…) We wanted to simplify it, but the preference [of the healthcare professionals] was to have all these functionalities in one device. Consequently, we ended up with a very complex and expensive device that no one wanted.’ Commercial technology developer A*
*2E*	*‘It seems like we’re making it [the technology] complicated together because they ask questions like “What do you want to see?” And when we provide an extensive list, they respond with additional questions on that list, and it goes on like that.’ Occupational therapist B*
*2F*	*‘I encountered an individual in a wheelchair who could not use their arms. They wanted to modify their wheelchair so that they could independently control the footrest. The healthcare professional communicated this request to the technology developer, which resulted in a modification—a small flip pin at the back. Everyone who saw this guy or talked with him would know that he couldn’t flip that switch.’ Professor, Applied Sciences*

#### III. Value proposition

This domain emphasises the importance of clearly understanding who will benefit from the technology and how it will generate value for them. Valuation is delineated into demand-side valuation (i.e., value for healthcare professionals, patients, and the health system) and supply-side valuation (i.e., value for technology developers). Tables 6 and 7 present representative quotes from this domain.

**Table 6:** Selected stakeholder quotations regarding the demand-side valuation within the value proposition

Identifier	Quote
3A	*‘I don’t know whether that [not having the capacity to meet demand] will happen. Perhaps we need to work more in groups, and with that, we can solve it.’ Physical therapist C*
3B	*‘It’s very simple. If we continue like this for the next 30 years, it’ll [healthcare] be too expensive and we won’t have enough people for it [delivery of healthcare services]. So, things must change.’ Physical therapist A*
3C	*‘We see a significant challenge in our healthcare system, which revolves around the cost, accessibility and quality. (…) Accessibility is already under immense pressure, and we expect that this will further intensify in the future (…) We think that innovation can help with this.’ Health insurer A*
3D	*‘The technology must truly add value compared to practical actions. In my opinion, practising how to crack eggs and pouring milk in a VR environment does not seem to be more valuable than doing it physically.’ Occupational therapist C*
3E	*‘I was talking with a board member of a care organisation, and they mentioned they would rather choose warm care over cold care (…). However, it’s not about warm care versus cold care or, as I call it, care facilitated by technology, it is care facilitated by technology versus no care because people will be unable to provide that care in the future and we can already demonstrate that.’ Strategist, sustainable healthcare*
3F	*‘A recent study suggests that all robotic technology for individuals with a neurological condition in the upper extremity is entirely pointless. They compared 1 h of therapy with a robot to 1 h of therapy with a therapist. Yeah, I don’t need a study for that, of course, the one with the therapist will be as good as or slightly better than the one with the robot. But that is comparing apples and oranges, it’s not about replacing healthcare professionals. It is about efficiency. With that robot, I can treat four people instead of one.’ Commercial technology developer A*

**Table 7:** Selected stakeholder quotations regarding the supply-side valuation within the value proposition

Identifier	Quote
*4A*	*‘Industry should decide to make a product out of it [the technology developed by universities]. They should bring the technology to the market and organise everything around it [certificates].’ University technology developer*
*4B*	*‘When developing new technology within research programmes, ambitious plans are often necessary to secure funding. For instance, in the case of a training device for arm exercises for stroke patients, a basic and straightforward design may not attract funding. However, projects that incorporate features such as feedback from the device or the involvement of therapists who must provide direct input stand a better chance of securing funding.’ Professor*
*4C*	*‘The rehabilitation market is quite small. That’s why we’re now being cautious and considering whether we can make money from the product before we decide to commercialise it.’ Commercial technology developer A*

##### Demand-side valuation

With the exception of some healthcare professionals (Q*3A*), all stakeholders acknowledged the value of using technology to address the considerable challenges of maintaining care accessibility within the Dutch healthcare system (*Q3B, C*). Some healthcare professionals perceived technology as valuable only when it exceeded their capabilities (*Q3D*). Strategy experts, management and innovation staff members, and developers mentioned that this comparison is common but unfair. Strategy experts and management and innovation staff members felt that comparing technology-facilitated care with the absence of care would be more appropriate, given the challenges of accessibility and anticipated future limitations of conventional care provision. By ‘absence of care,’ they meant scenarios in which capacity is insufficient to meet the demand (*Q3E*). Alternatively, they suggested comparing the number of patients that technology and healthcare professionals can assist in a 1-h period (*Q3F*).

##### Supply-side valuation

Commercial developers were noted by university developers to be responsible for the commercialisation of technology developed at universities (*Q4A*), whereas developers and strategic experts highlighted differences in the interests of commercial and university developers. One strategic expert observed that universities, driven by better chances of securing funding, often strive to develop highly innovative technologies targeting specific groups (*Q4B*). However, commercial developers may not be interested in these types of technologies, as the demand for them can be limited. Especially due to the limited size of the rehabilitation market, they emphasised the need for caution when introducing new technologies, as creating profitable products from them is challenging (*Q4C*).

#### IV. Adopter system

Within the adopter system, which involves the end-users, the implementation of technology is notably influenced by individuals’ perspectives on the technology. See Table 8 for representative quotes. People who had strokes and healthcare professionals expressed concerns regarding technology use in the home setting. The former voiced apprehensions about the quality of feedback, emphasising the importance of healthcare professionals’ guidance during exercise performance. They expressed that they expected to receive physical touch from healthcare professionals during therapy sessions and felt insecure about their exercise abilities (*Q5A*,* B*)*.* Healthcare professionals also expressed reservations regarding this patient group’s capabilities, placing greater emphasis on their proficiency in technology use than on their exercise abilities. They were concerned primarily about the possibility of errors during device setup or maintenance, which could result in injury. Additionally, they feared that patients might surpass the recommended training levels, thereby risking muscle overload (*Q5C*). Another concern mentioned by healthcare professionals was the possibility of an increasing workload. They noted that addressing patients’ concerns, responding to questions, and performing the required exercises within the limited 30-min timeframe of outpatient appointments was challenging, leaving them little or no time to review data or engage with the technology (*Q5D*)*.* However, management and innovation staff members emphasised that technology could be leveraged, such as with the use of online modules to create opportunities for professionals to optimise their time and perform additional tasks (e.g., engage with the technology) or to engage in more in-depth discussions during outpatient appointments (*Q5E*)*.*

**Table 8:** Selected stakeholder quotations regarding the adopter system

Identifier	Quote
*5A*	*‘For instance, a physical therapist might give a quick jab to your back, prompting you to stand up straight. This can’t be done through a screen.’ Person who had a stroke B*
*5B*	*‘But what if I perform the exercise incorrectly, and I don’t understand how to correct myself? Who will help me then?’ Person who had a stroke A*
*5C*	*‘Suppose you use VR combined with an exoskeleton. How many patients can install it correctly and take care of it? Besides, how do we prevent patients from overburdening themselves? How do we prevent them from experiencing shoulder pain after using it for 30 min instead of the suggested 15 min?’ Occupational therapist B*
*5D*	*‘It requires time. I’m already noticing that with the exercise portal that we currently have. It takes a lot of time, and time is something you hardly have. During outpatient care sessions, we only have half an hour with the patient. During that time, we must address questions and practise exercises, leaving minimal time to actively engage with the portal or review the activities.’ Occupational therapist B*
*5E*	*‘If professionals promote the digital modules, they’ll have more time available for other tasks. Numerous digital modules contain information provided by healthcare professionals. If individuals read these modules beforehand, there’s no need to repeat the same information, enabling a more thorough discussion during the appointment.’ Innovation programme manager*

#### V. Organisation

Some strategic experts, health insurers, and healthcare professionals highlighted that rehabilitation centres often overlook crucial aspects when implementing new technologies, including impacts on work processes, compatibility with the patient group, and the willingness to adopt the technology (*Q6A*,* B*). This oversight has been identified by these stakeholders as a reason for technology abandonment. Moreover, one strategic expert mentioned that management and innovation staff members often do not adopt specific implementation methodologies or tools when introducing new technologies (*Q6C*). However, management and innovation staff members did mention the use of some implementation strategies, such as engaging in conversations with healthcare professionals, emphasising the value of the technology through information channels, employing champions for promotion, and providing training in technology use (*Q6D*). Representative quotes from this domain are shown in Table 9.

**Table 9:** Selected stakeholder quotations regarding the organisation

Identifier	Quote
*6A*	*‘It is not the case that if we reimburse it, it automatically gets implemented. So, in many instances, these things just end up on the shelf. (…) because there hasn’t been any thought about how they actually want to implement it, whether people are willing to work with it, whether clients are willing to use it.’ Health insurer A*
*6B*	*‘For instance, a robotic assistive device that was purchased recently. Individuals with hemiplegia always walk with an asymmetrical centre of gravity. This is logical because one leg is not functioning well. However, the device was not designed with this consideration in mind (…). The complaint from our management is “You aren’t using that thing”. That’s correct; if you had listened to us, you wouldn’t have purchased it. Or, at the very least, you would have understood that we are not enthusiastic about using that device.’ Physical therapist C*
*6C*	*‘They don’t use methodologies, and if they do anything, they do what their predecessors used. I always say, well, it’s like they’re a carpenter; they all have a hammer, and whether it is dealing with a screw, a nail or a bottle, they insist on solving every problem with that hammer.’ Professor, Applied Sciences*
*6D*	*‘We distribute an A3-sized paper that can be placed on desks. Currently, it centres around telerehabilitation, offering tips for situations such as technical difficulties. The paper features helplines for both employees and clients. Additionally, we’ve designated “digicoaches”, who arehealthcare professionals. They play a crucial role in guiding colleagues through the process, recognising the significant impact when information is shared by a peer rather than directly from us [management].’ Innovation programme manager*

#### VI. Wider system

Within the wider system, the analysis revealed challenges within the political system (i.e., laws and regulations) and the economic system (i.e., financial system).

##### Political system

Innovation and management staff members, developers, health insurers, and the National Health Care Institute representative mentioned that the broader institutional context impacts the implementation of technology. For example, developers noted that strict regulations pose challenges to technology commercialisation and reimbursement. The Medical Devices Regulations and the Health Insurance Act were frequently referenced in this context. Developers pointed out that compliance with these requirements demands substantial time and resources (*Q7A*)*.* To offset these investments, commercial developers indicated that they must raise the costs of products, potentially diminishing their appeal to potential buyers (*Q7B*)*.* Health insurers acknowledged the challenge of receiving reimbursement for technologies but stated that they were bound by law (*Q7C*)*.* Table 10 provides representative quotes regarding the political system.

**Table 10:** Selected stakeholder quotations regarding the laws and regulations in the political system within the wider system

Identifier	Quote
*7A*	*‘Before it’s certified, my product will become significantly more expensive because the certification process costs a fortune. Instead of costing 100 euros, for example, it would be around 350 euros, and hardly anyone would buy it for that price.’ Commercial technology developer A*
*7B*	*‘This technology is a no-brainer if you read the literature (…) We now have to do a cost-effectiveness study because health insurers focus on the state of science and practice. However, this means it’ll take another five to six years before we can benefit from this innovation.’ Commercial technology developer B*
*7C*	*‘Everything covered by the basic insurance [Health Insurance Act] must adhere to the current state of science and practice. If we don’t know if that’s the case, we can’t reimburse it.’ Health insurer B*

##### Economic system

In the Netherlands, healthcare funding centres on diagnosis treatment combinations (DTCs), which represent the costs associated with specific diagnoses and the care pathways taken for specific medical conditions. Health insurers and the National Health Care Institute representative stated that rehabilitation centres are responsible for decisions about whether to incorporate technology into care pathways and the specific technologies to be used, as well as the associated costs (*Q8A*)*.* Innovation and management staff members, however, highlighted the challenge of bearing this financial responsibility, especially considering the ongoing financial difficulties that many centres are facing (*Q8B*)*.* They raised the prospect of a separate funding stream for technologies. According to the National Health Care Institute representative, the health insurer has the authority to decide this matter (*Q8C*)*.* Nevertheless, health insurers emphasised that rehabilitation centres remain accountable for the associated costs, as they are already covered under the DTC code. Despite this, they mentioned the availability of pilot programme funding, transformational funds, and subsidies to support the implementation of technology without drawing upon the rehabilitation centres’ budgets and noted that the centres are responsible for applying for this funding. Management and innovation staff members mentioned that they sometimes accessed these funds for technology implementation, but expressed uncertainty about continued funding, given the need for reapplication (*Q8D).*

In addition, innovation and management staff members pointed out that the existing financial system is structured to keep patients in rehabilitation centres. As compensation is provided exclusively for time spent with patients (*Q8E*), cases in which patients are discharged early or attend outpatient clinics less frequently may fall into less expensive DTC categories, creating financial disadvantages for the centres (*Q8F).* For representative quotes regarding the economic system, see Table 11.

**Table 11:** Selected stakeholder quotations regarding the financial system within the economic system of the wider system

Identifier	Quote
*8A*	*‘We’re talking about generic technology, then it has to come from the budget of the rehabilitation centre and therefore from the DTC (…). Why is this the case? Suppose a rehabilitation centre, for instance, decides to install an additional streetlamp at the entrance to enhance safety and prevent falls. In such instances, the associated costs, including these types of general expenses, are covered under the DTC code, and therefore, must be financed from the centre’s budget.’ Health insurer D*
*8B*	*‘But we do face quite a few challenges because we’re experiencing some financial difficulties, so we really have to keep asking for attention to keep it on the agenda.’ Innovation programme manager*
*8C*	*‘The therapist is entirely free to incorporate technology into their treatment. However, the decision about whether there should be a specific claim for it rests with the health insurer.’ National Health Care Institute representative*
*8D*	*‘You do have transformation funds. But we used the Stimulus Scheme for E-Health at Home, which allowed us to train many people and bring attention to the exercise portal. In 2024, there will be another round of this scheme, and we hope to obtain it again. Without these funds, implementation is challenging, as it requires time. Having compensation for it certainly makes the process easier.’ Innovation programme manager*
*8E*	*‘You simply get reimbursed for what you directly provide (…) So, you are encouraged to treat patients at the rehabilitation centre, because in that way you earn more money, because then you fall into a different category [higher DTC]. It completely contradicts the idea of earlier discharge.’ Innovation manager*
*8F*	*‘Let’s say I receive a directive from higher authorities to ensure that we implement more blended care, for example. If that works well, we might end up falling below the DTC threshold, meaning we are getting cheaper DTCs. Then they’ll say “Hey, what have you done?” That was not the intention, less income for us.’ Strategical innovation advisor*

#### VII. Fragmentation of responsibilities across all domains

This study identified a new theme, encompassing and affecting all theoretical domains of the NASSS framework: fragmented collaboration among stakeholders due to a lack of shared responsibility. In the development stage, technology developers emphasised the need for co-design with healthcare professionals to ensure the alignment of technologies with actual care delivery and to meet user groups’ preferences and needs. Conversely, healthcare professionals stated that developers should bear the primary responsibility for creating technologies that align with these aspects (Table 5). Similarly, in the valorisation stage, the interests and values of commercial technology developers and university technology developers were found to be misaligned, leading to a lack of shared responsibility and obstructing the commercialisation of such rehabilitation technologies (Table 7). Additionally, during the implementation stage, we observed a fragmentation of responsibilities. Rehabilitation centres were deemed responsible for technology implementation in the home setting but were influenced by challenges in the technology domain (Table 5*)* and the adopter system (Table 8)*.* Finally, fragmentation was also observed in the wider system (Table 11). This stems from the complex interconnectedness of the health system and economic system, which may be constrained by contradictory norms and misaligned values, such as the reimbursement model employed by the Dutch healthcare system. The common denominator across these challenges highlights the lack of cross-system boundaries and role clarity among stakeholders.

## Discussion

### Principal findings

In our effort to enhance the implementation of rehabilitation technologies in home settings, we explored stakeholders' perspectives on existing integration challenges. Nine factors were identified in six of the seven NASSS domains. In addition, the findings revealed a tenth factor, namely the fragmentation of responsibilities among diverse stakeholders. Tasks such as technology development, commercialisation, and implementation at rehabilitation centres were dispersed, with ill-defined responsibilities regarding development and implementation processes.

### Challenges across six NASSS domains

In the condition domain, participants recognised the complexity of stroke and the challenges that individuals encounter post-stroke, such as impaired motor function, cognitive decline, and language incapacities. The foremost concerns voiced by healthcare professionals centred on cognitive decline and language incapacities, as these participants foresaw potential complications in the use of home-based rehabilitation technologies. This perspective aligns with the findings of Braakhuis et al. [21] and Li et al. [15], underscoring its prevalence among healthcare professionals and the importance of considering these factors when developing home-based rehabilitation technology. If cognitive decline and language incapacities are not considered, the technology may not be suitable for supporting home-based rehabilitation, as patients may struggle to operate it independently.

In the technology domain, healthcare professionals, strategic experts, and management and innovation staff members observed that developers tend to create technologies without sufficient consideration of the care delivery process and the specific needs of end-users, resulting in technology abandonment. This perspective is consistent with the findings of Mitchell et al. [18]. Management and innovation staff members and strategic experts attributed this lack of consideration to developers’ lack of firsthand experience and knowledge. Developers acknowledged this gap and emphasised the importance of co-designing technology with healthcare professionals to address this, a viewpoint supported by Mitchell et al. [18]. Additionally, strategic experts highlighted the need to involve patients in the co-design process to ensure the technology meets the unique needs of the patients and is suitable for home use. Solely relying on healthcare professionals may be insufficient, as they may not fully understand or effectively communicate patients’ needs to developers. Therefore, the development of home-based rehabilitation technology should involve collaboration among developers, healthcare professionals, and patients.

In the value proposition domain, all stakeholders except some healthcare professionals acknowledged the value of home-based rehabilitation technology use to address the considerable challenges of maintaining care accessibility within the Dutch healthcare system. However, some healthcare professionals perceived technology as valuable only when it exceeded their own capabilities. Similar comparisons between care provided by healthcare professionals and that facilitated by technology are reported in the scientific literature [10, 11]. Nevertheless, strategy experts, management and innovation staff members, and developers argued that the evaluation of technology's value in this manner was unfair, especially given the accessibility challenges. They suggested that a more appropriate approach would be to compare technology-facilitated care with the absence of care, as resources are not expected to meet future needs. In this perspective, the focus is on improving efficiency rather than on improving effectiveness. To our knowledge, this perspective has not been prominently addressed in previous research. However, a shift in perspective for both home-based and clinic-based rehabilitation technologies could better illustrate their value. Thus, we opt for an approach that prioritises efficiency when assessing their value. Moreover, the main motivation for the commercialisation of university-developed technologies for (home-based) rehabilitation is financial gain, as confirmed by Siegel et al. [35]. Nonetheless, according to commercial developers and strategic experts, creating financially viable products from university-developed technologies in the rehabilitation market is challenging. This discourages further development, investment, and commercialisation of these technologies. As a result, the implementation of these technologies, both for home-based rehabilitation and in clinical settings, is hindered.

In the adopter system domain, people who had strokes emphasised the importance of in-person interaction with healthcare professionals in a clinical setting, believing that a hands-on approach was essential for effective training. This perspective aligns with the findings of Brouns et al. [23]. Cranen et al. [36] suggested that, for chronic pain patients, the perceived importance of a hands-on approach in a clinical setting was linked to a lack of familiarity with the technology in the home setting, and that this perception might shift with increased experience. Further investigation is needed to determine whether a similar pattern exists among individuals who had a stroke. In addition, healthcare professionals in this study expressed concerns about implementing rehabilitation technology for home-based rehabilitation. They noted that their workloads left little time for tasks such as data interpretation and setting up exercise modules. As a result, they were worried that adopting such technologies would increase their workload. Li et al. [15] reported similar concerns. Furthermore, some healthcare professionals raised concerns about patient safety, as patients would be using the technology without direct supervision at home. They worried that patients did not have the capabilities to use the device properly, which could lead to misuse and increase the risk of injury. This perspective is consistent with the findings reported by Braakhuis et al. [21] and Brouns et al. [23]. However, Brouns et al. [23] suggested that healthcare professionals might underestimate patients' capabilities, as some patients, initially deemed unsuitable for certain home-based technologies, were able to successfully engage with and benefit from them.

In the organisational domain, strategic experts, health insurers, and healthcare professionals pointed out that rehabilitation centres often overlook important factors when implementing new technologies, both in clinical and home settings. These factors include the impact on work processes, compatibility with the patient group, and the willingness of staff and patients to adopt the technologies. Additionally, participants noted that rehabilitation centres frequently favour practical approaches over specific implementation methodologies or tools when introducing new technologies. Thakur et al. [37] confirmed the preference of management staff for more practical approaches during the implementation of healthcare innovations. Phillips et al. [38] suggested that this preference could be explained by a lack of prior experience or familiarity with theories of implementation and behaviour change, which would cause people to feel uneasy and hesitant in their use. Further research is required to confirm this hypothesis.

The system domain includes laws, regulations, and financial systems. Developers highlighted challenges in commercialising and securing reimbursement for technologies used in clinical and home settings due to the Medical Devices Regulations and the Health Insurance Act. Compliance with these regulatory requirements demands significant investments of time and resources, as substantiated by Agyei et al. [39], and presents an additional challenge in bringing low-cost technology to the market. Health insurers acknowledged the challenges associated with the application of regulations, specifically the Health Insurance Act, but stated that they were bound by law. Furthermore, health insurers and the National Health Care Institute representative assigned the responsibility for costs associated with technology to the rehabilitation centres. However, management and innovation staff members considered separate funding to be essential. Brouns et al. [22] reported similar findings. Moreover, the participants felt that the care compensation model provided a perverse incentive. As compensation is based exclusively on time spent with patients, cases in which patients are discharged early or attend outpatient clinics less frequently fall into a less expensive DTC category, thereby creating financial disadvantages for rehabilitation centres. As a result, rehabilitation centres are hesitant to implement technology that supports home-based rehabilitation or to adopt home-based rehabilitation practices in general. Previous research has supported this observation within the healthcare system, emphasising the incentive to maintain care intensity above a specific threshold to increase revenue [40].

### Implications and future directions

This study observed fragmented collaboration among stakeholders. During the development and implementation stages, responsibility was transferred like a baton in a relay race, moving from university developers to commercial developers and then to rehabilitation centres. This handoff reflected a lack of shared responsibility, with each group concentrating solely on their own tasks.

To the best of our knowledge, this finding has not been previously reported in studies concerning home-based rehabilitation technology, nor has it been observed in research applying the NASSS framework to health technology. This may be due to the limited number of studies examining the entire design-to-implementation process involving all relevant stakeholders [15, 18–23]. While collaboration is not explicitly mentioned in the NASSS framework, our findings suggest that it is a prerequisite for the successful design, valorisation, and implementation of home-based rehabilitation technologies. Therefore, we recommend considering collaboration in the evaluation of these technologies, as it can help clarify why some of these technologies succeed and others do not. Nevertheless, further research is needed to validate whether collaboration genuinely enhances the evaluation and to assess its relevance in other contexts.

Our findings also underscore the need for enhanced stakeholder alignment, which can be addressed through relational coordination theory [41]. This theory is particularly relevant in interdependent, uncertain, and time-constrained contexts, like the implementation of home-based rehabilitation technology. It posits that effective collaboration hinges on high-quality, mutually reinforcing communication and relationships among stakeholders [41, 42]. Effective communication is characterised by timeliness, accuracy, frequency, and a problem-solving approach, while strong relationships are defined by shared knowledge, common goals, and mutual respect [41, 42]. Our study, however, revealed weak stakeholder relationships and suboptimal communication practices, including infrequent and delayed communication resulting from a focus on individual goals and unclear roles, leading to finger-pointing over responsibilities. To strengthen relationships and improve communication, structural, relational, and work process interventions could be employed [41]. For instance, the observed lack of role clarity could be addressed through work process interventions like the Responsible, Accountable, Consulted, Informed (RACI) matrix [41]. Nonetheless, further research is needed to explore the relationships and communication practices of stakeholders involved in implementing home-based rehabilitation technology in greater depth, as our study did not specifically address these aspects. We recommend using tools such as the Relational Coordination Survey [43] to assess relationships and communication practices and identify interventions for improvement in future studies.

Finally, the results indicate that the current lack of alignment of national policies and financial incentives fosters fragmentation. Therefore, we advocate that national policymakers take responsibility for developing policies that promote effective collaboration. A systems approach should be employed to analyse how stakeholders interact and define their roles within the healthcare system. This understanding is essential for developing policies that encourage collective accountability. For instance, policymakers could consider implementing a shared financial system to align the interests of all parties involved [41]. Further research should explore which policies could be most effectively aligned to incentivise collaboration among stakeholders in the implementation of home-based rehabilitation technologies.

### Limitations

Some limitations must be considered when interpreting the findings of this study. First, the interviewed stakeholders occasionally described technology in a general context rather than specifically in the home setting. This broader perspective may be attributed to their limited experience with the implementation of stroke rehabilitation technology in the home setting. However, it enhances the generalisability of the findings, allowing them to be applied beyond this context. Additionally, certain factors, such as laws and regulations are relevant to both home-based and clinical-based rehabilitation technologies. Thus, while this study focuses on home-based rehabilitation, some findings may also inform the implementation of rehabilitation technology in clinical settings.

Second, the representation of people who had strokes was limited, and informal caregivers were excluded from the study, which may have resulted in an incomplete understanding of all factors influencing implementation within the adopter system. The decision to exclude informal caregivers was based on their lack of experience with rehabilitation technology, as it is not yet widely implemented, thereby making it difficult for them to form meaningful expectations. Future research should specifically address the perspectives of informal caregivers with technological experience and include a greater number of individuals who had strokes to gain a more complete understanding of relevant factors.

Third, the suggestion to consider collaboration in the evaluation of the implementation of home-based rehabilitation technology is based on observations of its potential impact on the implementation process. However, we cannot definitively conclude that increased collaboration will lead to better outcomes, as this aspect was not examined. Future research should explore whether enhanced collaboration contributes to improved outcomes in the implementation of home-based rehabilitation technologies.

Fourth, the generalisability of the findings to other countries and health technologies may be limited, as the research was conducted solely among stakeholders in the Netherlands and focused on home-based rehabilitation technology. Future studies should investigate the experiences of stakeholders in diverse countries and across various health technologies to provide a more holistic understanding of the challenges and opportunities related to the implementation of health technologies.

Finally, we intentionally avoided focusing on a specific home-based rehabilitation technology in this study to provide a more comprehensive overview of the field. However, this choice meant that factors related to specific home-based rehabilitation technologies were not examined thoroughly, potentially limiting the depth of the analysis.

## Conclusion

This study shows that the sustainable implementation of home-based rehabilitation technology faces several challenges across various domains, including: (1) the unpredictability of stroke aftermath, (2) technology (mis)alignment with care delivery processes and target group preferences, (3) disparities in the assessment of technology’s value, (4) differences in commercial and university developers’ interests, (5) patient group capabilities, (6) perceived workload, (7) formal implementation plans in rehabilitation centres, (8) laws and regulations, and (9) the financial system. Effective collaboration among stakeholders is crucial to overcoming these challenges but is currently undermined by fragmented responsibilities. Thus, establishing alignment among stakeholders is essential. This requires clarifying roles and responsibilities. Moreover, national-level policy decisions that adopt a systems approach are necessary to align responsibilities and incentivise collaboration.

## Supplementary Information

Supplementary Material 1.

Supplementary Material 2.

## Data Availability

The data are available upon (reasonable) request.

## Abbreviations

NASSS Framework: Non-adoption, Abandonment, Scale-up, Spread, and Sustainability Framework
DTC: Diagnosis Treatment Combination
RACI matrix: Responsible, Accountable, Consulted, Informed matrix
