# Supplementary material for: The complexity of home-based rehabilitation technology implementation for post-stroke motor rehabilitation in the Netherlands
Source: BMC Health Serv Res. 2025 Jan 4;25:21. doi: 10.1186/s12913-024-12044-2 (PMC11699700; doi:10.1186/s12913-024-12044-2)
Supplement: Supplementary file 1 — Supplementary Material 1. [file 12913_2024_12044_MOESM1_ESM.docx]

**Topic-list**

| Topic | Questions |
| --- | --- |
| Background  **For all* | *For all:*   - Could you start by telling us a little bit about yourself, such as your gender, age, and occupation/education level |
| Condition  **Only healthcare professionals, people who had a stroke and developers* | *For healthcare professionals*   - Could you explain what a stroke entails? - Are there any comorbidities commonly associated with strokes?   *For developers*   - You are developing technology for individuals who have had a stroke. Could you tell me more about that?   *For individuals who had a stroke*   - Could you explain what a stroke entails? / How has the stroke affected you personally? - Do you have any other illnesses? |
| Technology  **For all* | *For developers:*   - Can you tell me about the technology you have developed technology?   *For healthcare professionals, innovation and management staff, health insurers, individuals who had a stroke, strategy experts, and National Health Care Institute members:*   - How familiar are you with rehabilitation technology? - Could you tell me more about the technology? - If you have used/seen it, what was your experience/ what did you think about it?   *For all:*   - What advantages does rehabilitation technology offer in home-based settings compared to traditional care methods? (e.g., no travel time, efficient, flexible, motivation, etc.) - What disadvantages might rehabilitation technology present in home-based settings compared to traditional care methods? (e.g., motivation, effective, etc.) |
| End-users system and value  **For all* | *For all:*   - How do you perceive the value of using rehabilitation technology in the home-setting? (e.g., improved quality, efficiency, enhanced collaboration) Why do you think these aspects are valuable? - What concerns do you anticipate that could affect its value? (e.g., quality, privacy, safety, time consumption, increased workload, necessity for significant delivery changes) Why do you believe these concerns are significant? - Are you inclined to prescribe/reimburse/ use rehabilitation technology in home-based settings? - If yes, what are the reasons for your inclination? Why do you consider these reasons important? - If no, what are the reasons for your hesitation? Why do you think these reasons outweigh the potential benefits? - How do you perceive others' attitudes toward the adoption of this technology—whether skeptical, negative, or positive? What factors contribute to these perspectives? Why do you think these factors shape their views? - Does their attitudes influence what you think about the technology? |
| Organisation  **Only healthcare professionals, management and innovation staff, strategy experts, developers, health insurers* | *For healthcare professionals, management and innovation staff, strategy experts, developers, health insurers*   - What are the barriers and facilitators to implementing rehabilitation technology within healthcare organisations? - Fit with the organization's norms and values, current work practices, changes in organizational structure, need for new personnel, new pathways and policies, workforce resistance, patients’ resistance, and budget constraints.   *For healthcare professionals, management and innovation staff*   - What incentives (financial or capacity) influence the decision to implement rehabilitation technology within an organization? - What are your previous experiences with implementing new interventions or technology? - What factors influenced their success or failure? (key players, management support, communication through meetings and emails, and training) |
| System  **Only healthcare professionals, management and innovation staff, strategy experts, developers, health insurers, National Health Care Institute* | *For healthcare professionals, management and innovation staff, strategy experts, developers, health insurers, National Health Care Institute*   - Which stakeholders or organizations are most important for the successful implementation of rehabilitation technology? Why do you think these are most important?   *For management and innovation staff, strategy experts,*   - Are there any local, state, or national policies or regulations that can impact the implementation of rehabilitation technology? - How do policies affect the implementation of rehabilitation technology? (Dutch policies regarding Integrated Care and Care at the Right Place)   *For health insurers and National Health Care Institute*   - What is the process for a developer seeking reimbursement for rehabilitation technology for home use? - When do you decide to reimburse? What is important? - Which stakeholders or organizations play a crucial role in the decision-making process to reimburse/include rehabilitation technologies for home settings in the basic package.   *For developers*   - Are there any local, state, or national policies or regulations that can impact the implementation of rehabilitation technology? - Is your technology reimbursed? - If yes, how did that process go? - If no, why not? What kind of difficulties have you experienced? |
| End  **For all* | **For all*  Before we end the conversation, I have one last question. We covered a lot of ground today—did I miss anything important that you would like to add? |
