# Supplementary material for: The complexity of home-based rehabilitation technology implementation for post-stroke motor rehabilitation in the Netherlands
Source: BMC Health Serv Res. 2025 Jan 4;25:21. doi: 10.1186/s12913-024-12044-2 (PMC11699700; doi:10.1186/s12913-024-12044-2)
Supplement: Supplementary file 2 — Supplementary Material 2. [file 12913_2024_12044_MOESM2_ESM.docx]

***Supplementary File 2. The Themes, Sub-Themes, Codes, and stakeholder reporting from Content Analysis***

| Theme | Sub-Theme | Codes | Reported by stakeholder |
| --- | --- | --- | --- |
| Condition | The unpredictability of stroke aftermath | Complex and unique needs of individuals who had a stroke (due to possible combination for motor, cognitive and language problems) | Healthcare professionals, technology developers and people who had a stroke. |
| Technology | Technology (mis)alignment with care delivery processes and target group preferences | Misalignment between the technology and healthcare delivery process | Healthcare professionals, strategic experts, management and innovation staff of rehabilitation centres |
|  |  | Misalignment in needs and preferences of the target group | Healthcare professionals, strategic experts, management and innovation staff of rehabilitation centres |
|  |  | Need for co-creation | Healthcare professionals, strategic experts and technology developers |
| Value Proposition | Disparities in the assessment of technology’s value | Value assessment criteria (comparing efficiency and effectiveness) | Healthcare professionals, strategic experts, management and innovation staff of rehabilitation centres, technology developers, health insurer and the representative of the National Health Care Institute |
|  | Differences in commercial and university developers’ interests | Interests (comparing profitability versus innovation) | Strategic experts and commercial and university technology developers |
| Adopter System | Patient group capabilities | Patients' low self-perceived capabilities | People who had a stroke |
|  |  | Healthcare professionals' perceived capabilities of patients are low | Healthcare professionals |
|  | Workload | Perceived workload | Healthcare professionals and innovation and management staff of rehabilitation centres |
| Organisation | Fromal implementation plan | Implementation plan and implementation strategies | Healthcare professionals, strategic experts, innovation and management staff of rehabilitation centres and health insurers |
| Wider System | Laws and regulations | Medical Device Regulation (increased production cost) | Technology developers |
|  |  | Health Insurance Act (Scientific rigour needed for reimbursement cost resources and time) | Technology developers and health insurers |
|  |  | Reimbursement rehabilitation technology | Health insurers and the representative of the National Health Care Institute |
|  | Financial system | Reimbursement providing care (disincentives home-based technologies) | Management and innovation staff members |
| Collaboration Among Stakeholders | Fragmented responsibilities | Unclear role boundaries among stakeholders throughout the implementation process and the absence of a structured responsibility framework. | Healthcare professionals, strategic experts, technology developers, management and innovation staff of rehabilitation centres, health insurers and the representative of the National Health Care Institute |
